# Japan can learn from the Swedish primary care doctor fostering system

**DOI:** 10.1002/jgf2.197

**Published:** 2018-07-28

**Authors:** Takashi Watari, Masahiro Hirose, Patrik Midlöv, Masanobu Okayama, Hiroo Yoshikawa, Kazumichi Onigata, Mikio Igawa

**Affiliations:** ^1^ Postgraduate Clinical Training Center Shimane University Hospital Shimane Japan; ^2^ Faculty of Medicine Department of Community‐based Health Policy and Quality Management Shimane University Izumo‐shi Japan; ^3^ Center for Primary Health Care Research Lund University Lund Sweden; ^4^ Division of Community Medicine and Medical Education Graduate School of Medicine Kobe University Kobe Japan; ^5^ Department of Internal Medicine Division of Neurology Hyogo College of Medicine Hyogo Japan; ^6^ Shimane University Hospital Shimane Japan

To the Editor

A new training program for primary care physicians in Japan started in 2018. However, there are many problems to overcome in the program, especially regarding the fact that the effective role of clinical education and clinical research of primary care physicians in the rural area in Japan are still not being sufficiently discussed. To investigate and improve the clinical research training system for primary care physicians in remote areas in Japan, we visited several primary healthcare centers and Lund University in Sweden between April 19, 2018, and April 26, 2018. In Sweden, there are seven medical schools that were established by their government, and the country has a total of approximately 2000 medical students. Japan has approximately 9000 medical students annually; however, the total population is about 9.5 million in Sweden.^1^ Although Sweden is referred to as the country with the most sophisticated social health system, an aging society with a declining birthrate is a huge problem as it is in Japan. In this survey, we concluded that primary healthcare centers have an important role not only with clinical manpower in remote areas but also as medical education sites for young physicians at the forefront of clinical research training. After the 18‐month initial postgraduate training period is finished, approximately 20% of the total trainees will select the primary care physician's special course (ST: special training) without duty or scholarships, which is considered to be the most important in the Swedish healthcare keeping.

Based on the recognition of the importance of high‐density training to become a skilled primary care physician, Swedish primary care physicians must complete at least 5 years of special training with at least 3 years at a primary healthcare center and at least 1.5 years of subspecial training, including internal medicine, obstetrics and gynecology, orthopedics, dermatology, psychiatry, pediatrics, otolaryngology, and ophthalmology, that specially focuses on each primary care setting.^2^ There is also a clinical scientific methodology course. Surprisingly, this course is mandatory and lasts at least 10 weeks; in the meantime, the trainees must try to acquire a scientific thinking ability to evaluate the appropriateness of their routine medical practice objectively; furthermore, they learn to create clinical questions and how to create a design and gather data, even if statistical analysis. Because many primary care physicians have PhDs in clinical research instead of basic laboratory science, they can conduct clinical research using the local PHC data and properly mentoring young trainees. In addition, primary care physicians who are interested in clinical research during the mandatory period can continue their research and take PhD courses (10% of total trainees) while working as clinicians at each PHC, and they can get strong support from the National Research School in General Medicine to improve the clinical research network among the primary care settings. Thus, we believe that there are many things to learn from Sweden's style about how to strengthen the Japanese skills of primary care physicians at university hospitals and training systems of clinical research in remote areas.^3^

## CONFLICT OF INTEREST

Authors T.W, M.H, P.M, M.O, and H.Y were supported by grants from Pfizer Health Research Foundation. The sponsor of the study had no role in the study design, conduct of the study, data collection, data interpretation, or preparation of the report.
